# Promotion of mitotic catastrophe via activation of PTEN by paclitaxel with supplement of mulberry water extract in bladder cancer cells

**DOI:** 10.1038/srep20417

**Published:** 2016-02-03

**Authors:** Nien-Cheng Chen, Charng-Cherng Chyau, Yi-Ju Lee, Hsien-Chun Tseng, Fen-Pi Chou

**Affiliations:** 1 Institute of Biochemistry, Microbiology and Immunology, College of Medicine, Chung Shan Medical University, Taichung City, Taiwan; 2 Research Institute of Biotechnology, Hung Kuang University, Taichung City, Taiwan; 3 Department of Pathology, Chung Shan Medical University Hospital, Taichung City, Taiwan; 4 Department of Radiation Oncology, Chung Shan Medical University Hospital, Taichung City, Taiwan; 5 College of Medicine, Chung Shan Medical University, Taichung City, Taiwan; 6 Clinical Laboratory, Chung Shan Medical University Hospital, Taichung City, Taiwan

## Abstract

Paclitaxel is a mitotic inhibitor used in cancer chemotherapy. Mulberry fruit is rich in phenolic compounds and flavonoids and exhibits chemopreventive activities. In this study, mulberry water extract (MWE) was used as a supplement to synergize with the effects of paclitaxel in the treatment of the TSGH 8301 human bladder cancer cell line. Treatment with paclitaxel combined with MWE (paclitaxel/MWE) enhanced the cytotoxicity of paclitaxel and induced severe G2/M arrest, mitotic catastrophe and subsequent apoptosis, as shown by MTT assay, HE staining and flow cytometry analyses. Differences in the expression and activation of Aurora A and Plk1between cells treated with paclitaxel/MWE and paclitaxel alone suggested that the combined treatment caused a defect in the early steps of cytokinesis. Paclitaxel/MWE decreased EEA1immunofluorescence staining and increased the expression of PTEN, indicating that the regimen inhibited the formation of the recycling endosome, which is required for cytokinesis. Paclitaxel/MWE also retarded tumor growth in a TSGH 8301 xenograft model via activation of PTEN and Caspase 3. These data demonstrated a synergistic effect on the anticancer efficacy of paclitaxel through MWE supplementation by promoting mitotic catastrophe through the activation of PTEN, providing a novel and effective therapeutic option for bladder cancer treatment strategies.

The incidence of urothelial carcinoma of the bladder is second in the urinary system only next to prostate cancer and occurs primarily in males^1^. Since the 1980s, the first-line chemotherapy for bladder carcinoma has been the combination regimen of methotrexate, vinblastine, doxorubicin and cisplatin (M-VAC) or gemcitabine and cisplatin^2^. However, previous studies have indicated a survival rate at 6 years of only 3.7% for patients receiving M-VAC therapy^3^, and the administration of M-VAC to elderly patients also presents considerable toxicity, including myelosuppression, nephrotoxicity, and neuropathy^4^. The most extensively studied second-line combination regimen is paclitaxel and gemcitabine, which has shown to be more effective and less toxic than M-VAC^5^.

Paclitaxel, an antitumor drug that demonstrates anti-tumor activity in human malignancies, is the first natural product shown to stabilize microtubules and, as a result, to interfere with the normal breakdown of microtubules during cell division^6^. Abnormalities in the dissociation of microtubules during mitosis or chromosome segregation can compromise cellular functions, reduce cellular fitness and lead to cell cycle arrest in G2/M phase. Specific regulatory proteins drive the cell cycle through M phase, such as the Cdc2/Cyclin-B1 complex; however, cells will remain at G2/M when this complex is not deactivated^7^. The correct segregation of chromosomes at anaphase is certified by the highly dynamic mitotic spindle. Successful formation of the mitotic spindle and the subsequent completion of cytokinesis is ensured by the action of three types of Aurora family kinases: Aurora A, B and C^8^. Aurora A, which is located in the pericentriolar material of centrosomes and regulates spindle formation, is particularly essential for accurate chromosome segregation^9^.

Mitotic catastrophe is one of the strategies used by higher eukaryotes to eliminate mitosis-incompetent cells^10^,^11^. The morphological changes associated with failed mitoses and that serve as the most prominent characteristics of mitotic catastrophe are micronucleation and multinucleation, which are the outcomes of chromosomal breaks and deficient karyokinesis^11^. It has been proposed that mitotic catastrophe is an oncosuppressive mechanism preceding apoptosis, necrosis or senescence^10^. Therefore, the blockage of mitotic catastrophe would promote tumorigenesis and cancer progression, whereas its induction would presumably provide a successful therapeutic outcome. Nonetheless, the molecular mechanism of mitotic catastrophe is poorly understood. The characteristic multinucleation in mitotic catastrophe is the result of a defect in cytokinesis which is the final event of the cell cycle and is the process that divides one cell into two daughter cells^12^. During cytokinesis, a cleavage furrow containing actin, myosin and other proteins is formed at the cell equator, and then Golgi and early recycling/late endosomal membranes, which deliver various lipids and proteins needed for cytokinesis, cluster at both edges of the intercellular bridge^12^. Because early recycling endosomes are composed of phosphatidylinositol (3,4,5)-trisphosphate (PtdIns(3,4,5)P3 or PIP3), the dephosphorylation of PtdIns(3,4,5)P3 by the PTEN protein reduces the formation of early endosomes in the cytoplasm, and consequently jeopardizes cytokinesis^13^.

The use of natural products as auxiliary agents is an important trend in cancer research and has the goals of increasing the efficacy of present cancer treatment, lowering toxic effects and alleviating side effects. The mulberry is an important medicinal plant belonging to the Moraceae family, and several phenolic compounds and many flavonoids have been isolated from various parts of this plant^14^. In previous studies, it has been shown that flavonoids and other phenolic compounds have potentially beneficial effects, including anti-inflammatory, antibacterial, antiviral, and antioxidant activities^15^,^16^,^17^. In addition, flavonoid derivatives have been confirmed as effective and novel chemotherapeutic drugs in bladder cancer therapy^18^, and another study has provided evidence that flavonoids have synergistic effects in photodynamic therapy (PDT), improving treatment outcomes and reducing metastasis in T24 and MB 49 bladder cancer lines^19^.

In this study, we demonstrated that the cytotoxicity of paclitaxel was enhanced by MWE via promoting mitotic catastrophe through a mechanism in which increased PTEN activity reduced the formation of early endosomes and consequently influenced cytoplasmic membrane division in bladder cancer cells. These results suggested that the induction of mitotic catastrophe could be considered as an alternative strategy for cancer therapy and that MWE could be used as an auxiliary to enhance clinical cancer chemotherapy.

## Results

### Paclitaxel combined with MWE induced TSGH 8301 bladder carcinoma cell death by arresting the cell cycle at the mitotic phase

**Figure 1:**
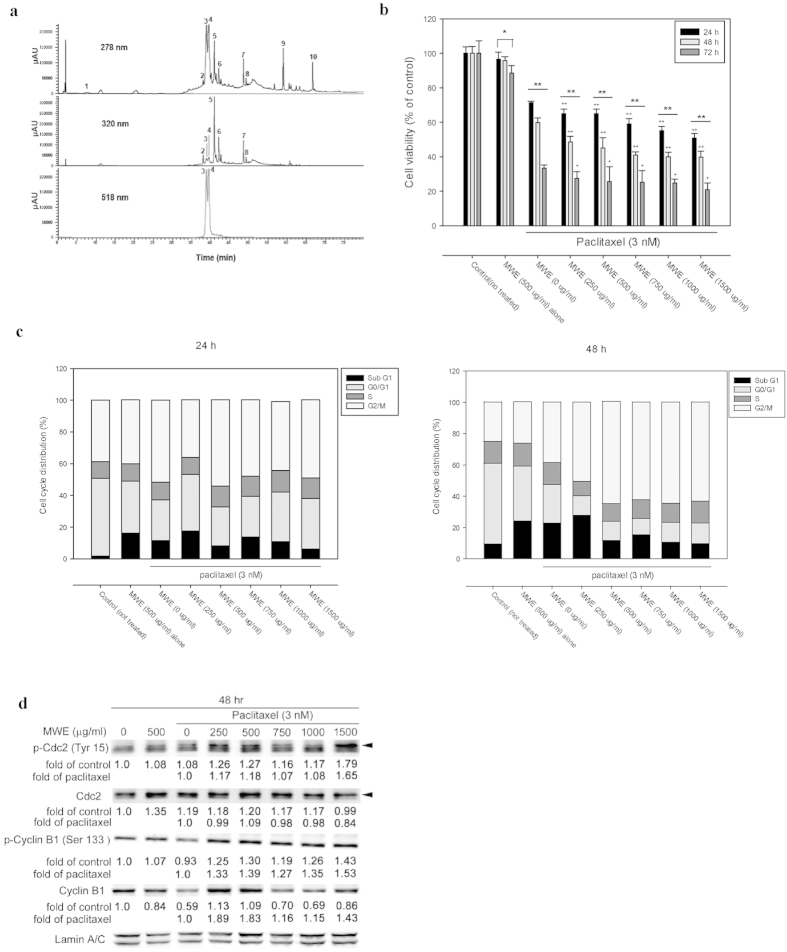
Paclitaxel combined with MWE induced TSGH 8301 bladder carcinoma cell death by arresting the cell cycle at the mitotic phase. (**a**) HPLC-DAD profiles of MWE. A gradient solvent elution system was used over 80 min with acetonitrile (containing 0.1% formic acid) and 0.1% aqueous formic acid at a flow rate of 0.2 ml/min. Detection is shown at 278, 320 and 518 nm. The numbering of the peaks refers to their identification, as shown in Table 1. (b) TSGH 8301 cells were treated with paclitaxel alone or combined with the indicated concentrations of MWE for 24, 48 and 72 hr before being subjected to the MTT assay for cell viability. The data are expressed as a percentage of control (not treated) and presented as the means ± SD. One-way ANOVA with post-hoc Dunnett’s test was used to calculate the *p* value for each dose treatment compared to paclitaxel alone, (^+^*p* < 0.05; ^+^^+^*p < *0.01) and between time points (^*^p < 0.05; ^**^p < 0.01). (**c**) TSGH 8301 cells were treated with paclitaxel (3 nM) and MWE (0–1500 μg/ml) and then subjected to cell cycle distribution analysis by flow cytometry at 24 hr and 48 hr. (**d**) Protein samples were prepared from different treatments at 48 hr and analyzed by Western blotting for Cdc2 and Cyclin B1. The numbers under each blot are the intensity of each band relative to that of the control (not treated) or paclitaxel alone. The blots were reprobed with an anti-Lamin A/C antibody to confirm equal loading of the samples. Arrow head indicated the band used for quantitation. The results shown are representative of three independent experiments with similar results.

The constituents of MWE prepared in this study were analyzed by HPLC-DAD-ESI-MS. The typical UV-Vis spectra of anthocyanins recorded at λ max 518 nm are shown in Fig. 1a, with obvious profile differences from those at 278 and 320 nm, which are presented as typical absorption spectra of phenolic compounds and flavonoids. The identities of the compounds were obtained by matching their molecular ions (m/z) obtained by LC-ESI-MS and LC-ESI-MS-MS with data from the literature, and the results are summarized in Table 1.

The cytotoxic effect of paclitaxel and MWE was determined in a bladder carcinoma cell line (TSGH 8301) by measuring cell viability with the MTT assay (Fig. 1b). Treatment of paclitaxel (3 nM) caused cell death in a time-dependent manner that was further enhanced by the combined exposure to various doses of MWE. The cytotoxicity of the combined treatment was dependent on both the MWE dose and the treatment duration. Similar results were obtained from the experiments using two additional bladder carcinoma cells lines (HT1367 and HT1197) (Supplementary Fig.-1). In contrast, MWE alone (0–1500 μg/ml) showed only a slight toxic effect (Supplementary Fig.-2).

To identify the possible death mechanism invoked by the combined treatment of paclitaxel and MWE, the cell cycle distribution of the treated cells was analyzed. As shown in Fig. 1c, paclitaxel at a dose of 3 nM increased the cell populations in the subG1 and G2/M phases, whereas MWE (500 μg/ml) alone increased the subG1 cell number but not that of G2/M. The percentage of cells in G1 phase was substantially lower in the TSGH cells exposed to MWE alone, paclitaxel alone and paclitaxel plus MWE compared to that of the untreated cells, especially at the 48 hr time point. This decrease in cells in G1 phase was accompanied by an apparent increase in the G2/M-phase population, with approximately 63% of the cells treated with paclitaxel and MWE (500 to 1500 μg/ml) found in the G2/M phase at 48 hr and only 25%, 27% and 40% for the control, MWE and paclitaxel groups, respectively. However, the subG1-phase populations of the MWE alone, paclitaxel alone and the combined groups with a low dose of MWE (250 μg/ml) were apparently higher than those of the combined treatments at higher doses of MWE (500 to 1500 μg/ml). To confirm the state of G2/M arrest induced by the combined treatment, the nuclear levels of Cyclin B1 and Cdc2 and their phosphorylation were examined by Western blotting at 48 hr after exposure (Fig. 1d). The results showed that paclitaxel alone induced Cdc2 and repressed Cyclin B1 compared to the control cells, with the addition of MWE to paclitaxel enhancing the amount of both Cyclin B1 and Cdc2 and their phosphorylated forms in the nucleus. These data indicated that the combined treatment of paclitaxel and MWE blocks the M phase transition because Cyclin B degradation and Cdc2 dephosphorylation are required for the cell to progress to the G1 phase^20^.

### Combined treatment of paclitaxel and MWE induced mitotic catastrophe in TSGH 8301 cells

**Figure 2:**
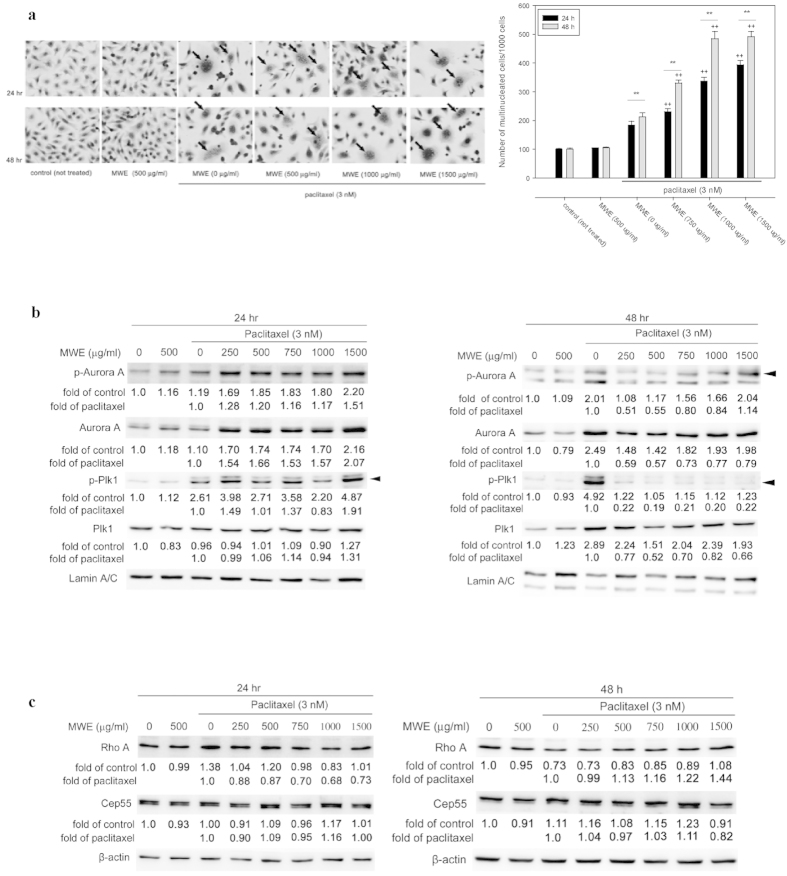
Paclitaxel combined with MWE induced multinucleation and affected mitotic catastrophe-related protein expression levels in TSGH 8301 cells. (**a**) TSGH 8301 cells were subjected to HE staining at 48 hr after the indicated treatments (left panel). The arrows point to the cells with multinucleation. Right panel, quantitation of the number of multinucleated cells/ 100 cells. (**b**) Nuclear extracts were prepared to assess Aurora A and Plk1. (**c**) Cytoplasmic lysates were prepared from TSGH 8301 cells treated with paclitaxel, MWE or both together at 24 and 48 hr and subjected to Western blotting analysis for the levels of Rho A and Cep55. The numbers under each blot are the intensity of each band relative to that of the control (not treated) or paclitaxel alone. The relative protein amounts were quantified, and the results are normalized to that of either β-actin (cytoplasm) or Lamin A/C (nucleus). Arrow head indicated the band used for quantitation. The results shown are representative of three independent experiments with similar results.

Multinucleation is the most prominent morphological trait of mitotic catastrophe, a form of cell death associated with abnormal mitosis. The data from HE staining revealed that a portion of the TSGH 8301 cells treated with paclitaxel, but not MWE, displayed multinucleation (arrows) at 24 and 48 hr (Fig. 2a). The combined treatment increased the number of multinucleated cells at both time points (Fig. 2a, right panel). Because the evidence that treatment with a combination of paclitaxel and MWE induced G2-M arrest and multinucleation in TSGH 8301 cells strongly suggested the involvement of mitotic catastrophe, the nuclear levels of Aurora A, whose expression and activation is involved in regulating the functions of the centrosomes, spindles and kinetochores required for proper mitotic progression, as well as its phosphorylated form, were analyzed by Western blotting. As shown in Fig. 2b, slight increases in Aurora A and phospho-Aurora A induced by paclitaxel were further enhanced by MWE at 24 hr. When the treatment duration was extended to 48 hr, paclitaxel caused a marked increase in the expression of Aurora A and its phosphorylated form, and the addition of MWE repressed the levels of both parameters in a dose dependent manner, with lower doses exhibiting higher repression. This observation indicated that the cells exposed to the combined treatment were able to undergo nuclear division but could not escape from M phase, a phenomenon that was different from that observed with paclitaxel alone. Both Rho A and Plk1 in the cytoplasm are implicated in the regulation of cytokinesis, which is the last step in M phase and marks the division of the cytoplasm of a parent cell into daughter cells after nuclear division. Biochemical inactivation or depletion of Rho A leads to cleavage furrow formation failure^21^, and overproduction of Plk1 results in multinucleation in mammalian cells, which is indicative of cytokinesis failure^22^. Cep55, a crucial factor required for abscission, the final step of cytokinesis, is recruited to the anaphase spindle by Plk1. The Western blotting data showed that Plk1 phosphorylation, but not the protein itself, was increased at 24 hr in the cells treated with paclitaxel alone and that both total Plk1 and its phosphorylated form were greatly elevated at 48 hr compared to the control cells (Fig. 2b). The combined treatment also induced the phosphorylation of Plk1 at 24 hr. However, Plk1 was dephosphorylated to a level comparable to that of the control at 48 hr, even though the total protein was also increased, indicating that the deactivation machinery was functional in the combined treatment groups but not in the paclitaxel alone group. In addition, the level of Rho A was induced by paclitaxel at 24 hr but was repressed at 48 hr compared to the control (Fig. 2c), and the addition of MWE counteracted the action of paclitaxel by dose dependently repressing the amount of Rho A at 24 hr and increasing it at 48 hr. The combined treatment with 1500 μg/ml MWE revealed levels of Rho A similar to those of the control cells at both time points. None of the treatments caused significant changes in the level of Cep 55 (Fig. 2c), and exposure to MWE (500 μg/ml) alone did not significantly affect the expression of any of the detected proteins. All these data suggested that the mechanism of G2/M arrest and multinucleation induced by paclitaxel is different from that of the combined treatment and that the defect with the latter might occur at the early steps of cytokinesis.

### Combined treatment of paclitaxel and MWE impaired early endosome generation in TSGH 8301 cells

**Figure 3:**
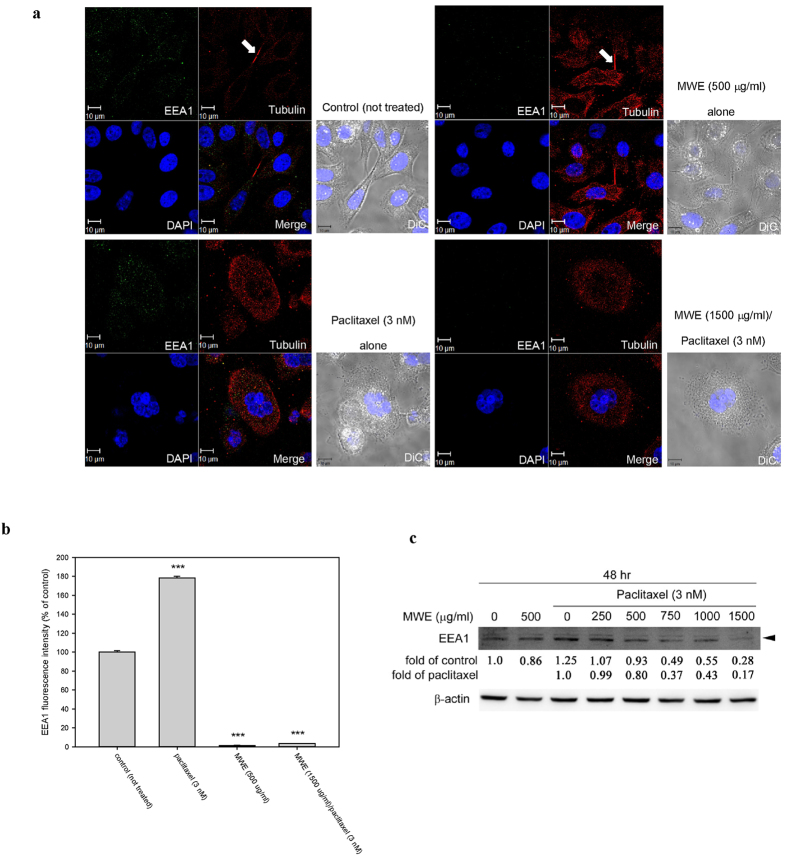
Combined treatment of paclitaxel and MWE impaired early endosome formation in TSGH 8301 cells. (**a**) Cells were treated with the indicated concentrations of paclitaxel and MWE for 48 hr and were then fixed, stained with an anti-EEA1 antibody (green), an anti-α-tubulin antibody (red) and DAPI (blue) and observed under confocal microscopy. Arrows indicate the formation of the midbody in dividing cells. (**b**) EEA1 levels in the cells were quantified, and the results are expressed in means ± SD as a percentage of the control (not treated). (**c**) Cell lysates were prepared from TSGH 8301 cells treated with paclitaxel, MWE or both together at 48 hr and subjected to Western blotting analysis for the levels EEA1. The numbers under the blot are the intensity of each band relative to that of the control (not treated) or paclitaxel alone. The relative protein amounts were quantified, and the results are normalized to that of β-actin. Student’s t-test was used to calculate the *p* value for each treatment compared to the control (indicates ^***^p < 0.001).

During cytokinesis, the cleavage furrow is assembled at the division site, which is generally at the cell equator, and various membrane organelles including recycling endosomes are clustered at the pericentrosomal region near the cleavage furrow, which is dependent on the activity of microtubule motors^23^. Early endosome antigen 1 (EEA1), which localizes exclusively to early endosomes, is important in endosomal trafficking to the pericentrosomal region^24^. Confocal microscopy was used to observe the location of EEA1 in TSGH 8301 cells treated with different regimens. As shown in Fig. 3a, typical midbodies with a tightly packed microtubule bundle at each of their cores connecting two daughter cells were observed in dividing control cells as well as in the cells treated with MWE alone (Fig. 3a, arrows). Conversely, such a structure was not found in the cells exposed to paclitaxel and the combined treatment. EEA1 was localized in the cytoplasm and at the center of the midbody, indicating the cellular position of the early endosome (green florescence), and the level of EEA1 was significantly enhanced by paclitaxel and was prominently repressed by MWE alone or in combination with paclitaxel (Fig. 3b). The results of Western blotting demonstrated that EEA1 was reduced in the paclitaxel/MWE treatment groups dose-dependently (Fig. 3c). These data suggested that MWE alone can inhibit the generation of early endosomes; thus, together with paclitaxel, which stabilizes the microtubule polymer and protects it from disassembly, MWE appears to block cytokinesis.

### Paclitaxel combined with MWE induced PTEN activation and expression and inhibited early endosome formation in TSGH 8301 cells

**Figure 4:**
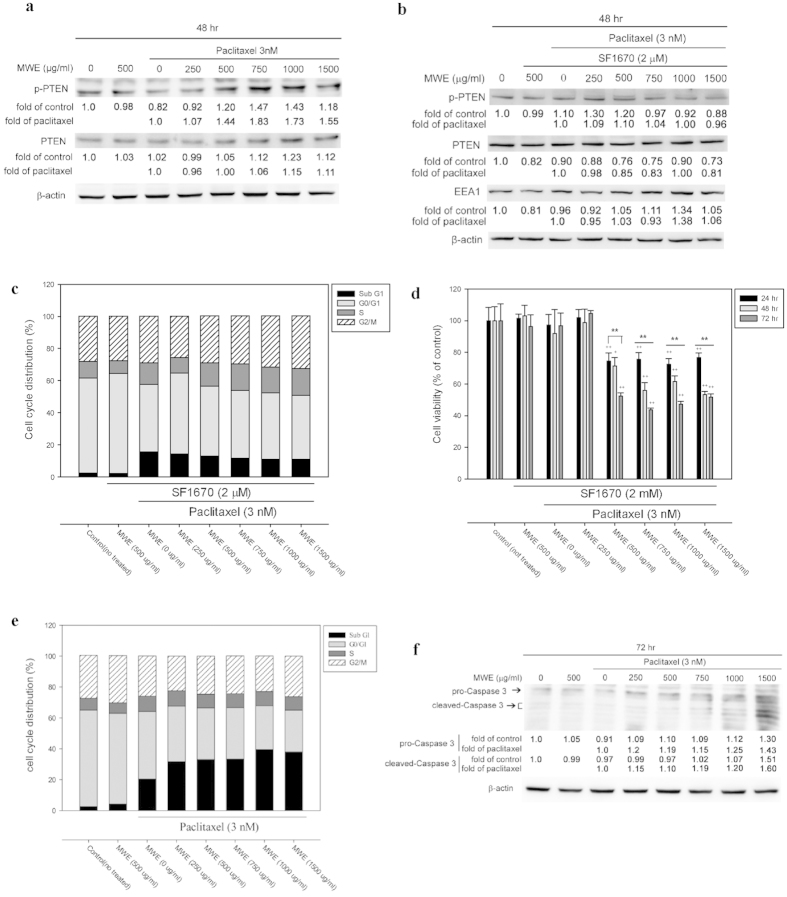
Paclitaxel combined with MWE induced PTEN activation and expression in TSGH 8301 cells. (**a**) Cytoplasmic lysates were prepared from TSGH 8301 cells treated with paclitaxel, MWE or both together at 48 hr and subjected to Western blotting analysis for the levels of PTEN. (**b**) TSGH 8301 cells were pre-treated with PTEN inhibitor SF1670 (2 μM) for 24 hr followed by paclitaxel, MWE or both together, as indicated. Cytoplasmic lysates were prepared and subjected to the detection of PTEN and EEA1 expression by Western blot analysis at 48 hr. (**c**) TSGH 8301 cells were pre-exposed to PTEN inhibitor SF1670 (2 μM) for 24 hr before treatment with paclitaxel, MWE or both together for 48 hr. The cells were harvested and subjected to cell cycle distribution analysis by flow cytometry. (**d**) TSGH 8301 cells were pre-treated with the PTEN inhibitor SF1670 (2 μM) and then treated with the indicated concentrations of paclitaxel, MWE or both for 24, 48 and 72 hr. The cells were harvested and subjected to an MTT assay for cell viability analysis. The data are expressed in means ± SD as a percentage of the control (not treated). One-way ANOVA with post-hoc Dunnett’s test was used to calculate the *p* value for each treatment compared to paclitaxel alone, (^+^*p* < 0.05; ^+^^+^*p* < 0.01) and between time points (^*^p < 0.05; ^**^p < 0.01). (**e**) and (**f** ) TSGH 8301 cells were treated with paclitaxel, MWE or both together for 72 hr and then subjected to quantitation of the cell cycle distribution by flow cytometry and Western blotting analysis for the detection of Caspase 3. The numbers under each blot of (**a**), (**b**) and (**f** ) are the intensity of each band relative to that of the control (not treated) or paclitaxel alone. β-actin was used as the protein loading control. The results shown are representative of three independent experiments with similar results.

The tumor suppressor PTEN, a 3′ phosphoinositide phosphatase that converts PI(3,4,5)P_3_ to PI(4,5)P_2_, was originally identified as a negative regulator of phosphoinositide 3-kinase (PI3K) signaling, a main regulator of cell growth, metabolism and survival^25^. PI(3,4,5)P_3_ the major component of early endosomes, has recently been suggested to regulate cytokinesis, based on observations that PI(3,4,5)P_3_-endosomes are present at the midbody and that PI(3,4,5)P_3_ kinase (PI3K) inhibitors block cytokinesis and lead to the accumulation of multinuclear cells^26^. To investigate the role of PTEN in this study, Western blotting was used to detect levels of total PTEN and active-PTEN (phospho-PTEN) in TSGH 8301 cells treated with different regimens at 48 hr. Paclitaxel reduced the level of active-PTEN but not total PTEN as compared to the control cells (Fig. 4a), and treatment with MWE plus paclitaxel significantly increased PTEN phosphorylation and total PTEN to a lesser extent when compared to paclitaxel alone. The role of PTEN was further examined by pretreating TSGH 8301 cells with SF1670, a PTEN inhibitor, for 24 hr before treatment with the different regimens. Pretreatment with SF1670 repressed the enhancement of PTEN phosphorylation and total protein and released the suppression of EEA1 caused by the combined treatment at 48 hr (Fig. 4b, compared to Fig. 4a and Fig. 3b). At the same time, no significant G2/M arrest was observed in TSGH 8301 cells under different treatments, indicating that the cells had escaped from G2/M phase due to PTEN inhibition (Fig. 4c). The blockage of PTEN activity also repressed the cytotoxicity of paclitaxel and increased the viability of cells under the combined treatment at all three time points (Fig. 4d). These data confirmed that the addition of MWE in paclitaxel treatment induced PTEN expression and activation, interfering with the formation of early endosomes and subsequently impairing cytokinesis in TSGH 8301 cells. The consequence of mitotic catastrophe induced by the combined treatment was further explored by determining the cell cycle distribution at 72 hr. As shown in Fig. 4e, flow cytometry detected a relatively higher ratio of sub-G1-population cells in the combined treatment at 72 hr than in the paclitaxel group, suggesting that mitotic catastrophe would eventually lead to apoptosis. The apoptotic state of the cells was confirmed by dose-dependent increases in the levels of pro-Caspase 3 and its active form in the cells treated with paclitaxel and MWE (Fig. 4f). These data demonstrated that the combination of paclitaxel and MWE targets PTEN activity and leads to mitotic catastrophe and apoptotic cell death in TSGH 8301 bladder cancer cells.

### Paclitaxel in combination with MWE retarded tumor growth in a human bladder carcinoma TSGH 8301 xenograft model

**Figure 5:**
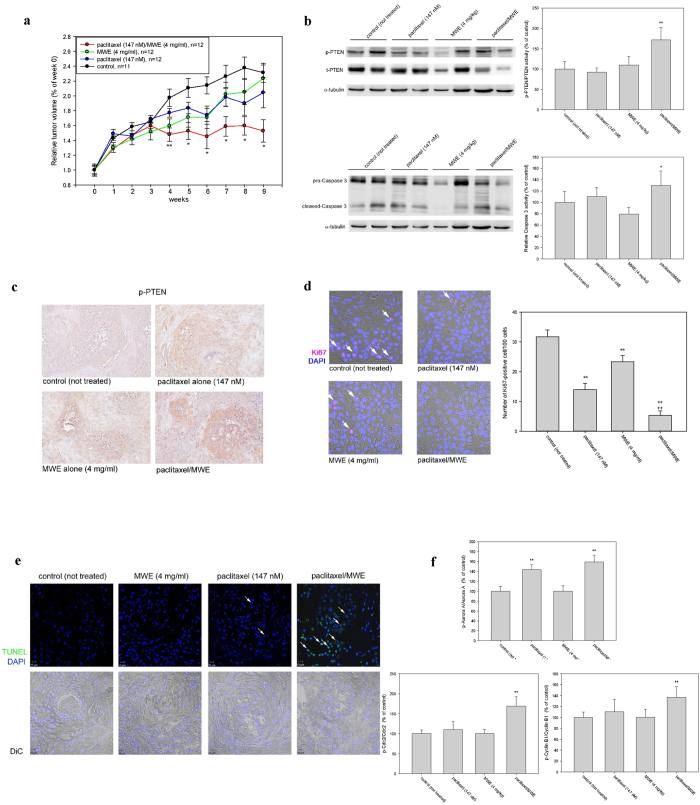
Paclitaxel in combination with MWE retarded tumor growth in a human bladder carcinoma TSGH 8301 xenograft model. (**a**) TSGH 8301 cells (1 × 10^7^ cells/mouse) were injected into the right inguinal region of a nude mouse to form tumor xenografts. When the tumor size reached approximately 250 to 300 mm^3^, the mice were randomly divided into 4 groups and received the following treatments: paclitaxel combined with MWE, MWE alone, paclitaxel alone and sterile deionized water (control group). Tumor size was monitored every week, and the results are expressed as the percentage of the size at week 0 (the day treatment started) for each group. (**b**) The levels of total (t-PTEN) and phospho-PTEN (p-PTEN) and Caspase 3 in the tumor specimens were determined by Western blotting and then quantified using β-actin as the protein loading control; the results are expressed as a percentage of the control. (**c**) Immunohistochemical examination of p-PTEN in the tumor sections obtained from the indicated treatment. (**d**) and (**e**) Fluorescent immunohistochemical detection of Ki67 and TUNEL examination in the xenografts obtained from the indicated treatment. (**f** ) Western blotting analysis of the levels of Cyclin B1, Cdc2 and Aurora A in xenograft tumors. Arrow indicates the Ki67 or TUNEL positive cells. One-way ANOVA with post-hoc Dunnett’s test was used to calculate the *p* value for each treatment compared to paclitaxel alone, (^+^*p* < 0.05;^+^^+^*p* < 0.01) at each time point (**indicates *p* < 0.01 and *indicates *p* < 0.05).

To further evaluate the anti-tumor effects of MWE combined with paclitaxel, an *in vivo* xenograft model by subcutaneous inoculation of TSGH 8301 cells into nude mice was performed. Tumors were established approximately 20 days after implantation (mean volume, 450–500 mm^3^), and the animals were given paclitaxel (147 nM, once a week for 9 weeks) alone or in combination with MWE (4 mg/kg/day for 10 weeks) or MWE alone. The average tumor volume of the combined treatment group was significantly smaller than that of the control group starting at the 5th week and thereafter (Fig. 5a). By the end of the experiment, there was a 32% reduction in average tumor volume (813 ± 147 mm^3^) in the combined treatment group compared to the control animals (1182 ± 228 mm^3^). The average tumor sizes of the MWE alone and paclitaxel alone groups tended to be smaller than that of the control but did not show statistical significance (Fig. 5a). To assess whether the cellular mechanism of the combined treatment was also applicable in the xenograft model, Western blotting was used to examine the tumor levels of total-PTEN, phospho-PTEN, pro-Caspase 3 and cleaved-Caspase 3. Although all the treatment groups did not show higher levels of these four factors than the control tumors (Fig. 5b), the tumors obtained from the combined treatment demonstrated significantly higher activities of both PTEN and Caspase 3, as shown by the ratios of phospho-PTEN to total-PTEN and cleaved-Caspase 3 to pro-Caspase 3 (Fig. 5b). Immunohistochemical examination also revealed a higher level of p-PTEN in the tumor section obtained from the combined treatment (Fig. 5c). Detection of Ki67 by immunohistochemistry and TUNEL (terminal deoxynucleotidyl transferase dUTP nick end labeling) in the xenografts demonstrated that paclitaxel/MWE treated tumors have less Ki67-positive cells and more TUNEL-positive cells, suggesting that the combined treatment has stronger toxicities than paclitaxel alone and MWE alone (Fig. 5d,e). The levels of Cyclin B1, Cdc2 and aurora A in xenograft tumors were examined by Western blotting. The data showed paclitaxel alone and MWE/paclitaxel induced p-Aurora A expression (Fig. 5f). Moreover, the combined treatment, but not MWE or paclitaxel alone, enhanced p-Cdc2 and p-Cyclin B1 levels. These data corresponded to the *in vitro* study. On the basis of these results, the finding that paclitaxel synergizes with MWE and significantly improves antitumor efficacy could have important clinical implications.

## Discussion

The mechanism of action of paclitaxel, a cytoskeletal drug that targets tubulin, is to stabilize the microtubule polymer and prevent it from disassembly, thus blocking the progression of mitosis. When the mitotic checkpoint is activated in a prolonged manner, cells will undergo apoptosis or reverse to the G-phase of the cell cycle without cell division^27^. Recent work has indicated that paclitaxel at a low concentration (10 nM) causes a mitotic block followed by aberrant mitoses, multiple micronuclei, and tetraploidy (mitotic catastrophe), which may be followed by apoptosis^28^. In the present study, 3 nM paclitaxel prompted relatively low levels of G2/M arrest and apoptosis in TSGH 8301 bladder carcinoma cells. Indeed, the presence of cells with multinucleation indicated the induction of mitotic catastrophe under this condition. Supplementation of paclitaxel with an extract of mulberry resulted in the arrest of approximately 60% of the cells in G2/M phase at 48 hr and 30–35% apoptotic cells at 72 hr, suggesting that the combined treatment increased the occurrence of mitotic catastrophe and led to apoptosis.

Plk1, a regulator involved in centrosome maturation, spindle assembly, sister chromatid cohesion, cytokinesis and recovery from DNA-damage-induced arrest, is activated and phosphorylated by the Bora-Aurora A complex. Plk1 activity is sustained in mitosis^29^. Our data showed that the levels of phosphorylated and total Plk1 and Aurora A protein returned to control levels after the combined treatment; these factors were accumulated in the paclitaxel alone group at 48 hr. These differential responses provide evidence supporting that the cells exposed to paclitaxel and MWE were able to complete nuclear division but did not continue to the ensuing cytokinesis, a mechanism that was different from that of paclitaxel alone.

**Figure 6:**
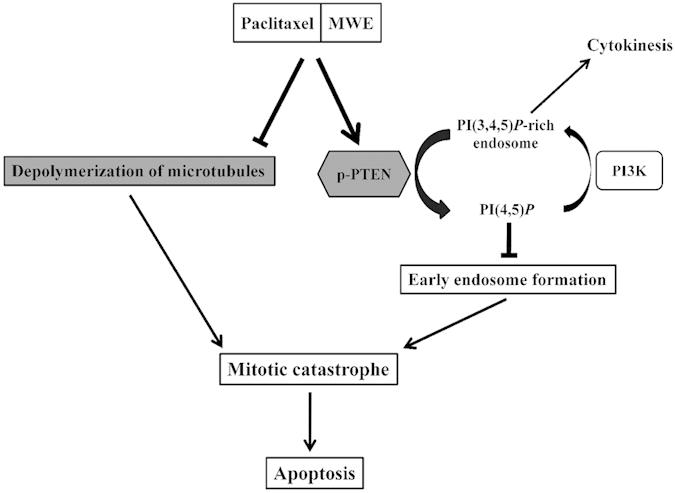
Schematic diagram of the proposed mechanism of the induction of mitotic catastrophe via PTEN activation by the combined treatment of paclitaxel and MWE.

Induction of programmed cell death, especially apoptosis, is one of the main strategies of current cancer therapy. The modulation of apoptosis through the manipulation of autophagy to induce cell death and inhibit protective autophagy also has therapeutic implications though remains an intensely debated concept^30^. Mitotic catastrophe has received increasing attention in recent cancer research because of its potential application in cancer therapy. The induction of mitotic catastrophe could be achieved by a heterogeneous group of stimuli via different mechanisms, such as DNA damaging agents^31^, depletion of centrosomal proteins^32^ and inhibitors of Aurora^33^. Studies of synergistic treatments inducing mitotic catastrophe have also shown convincing outcomes, such as using a PPAR γ inhibitor to enhance radio-sensitivity in human cervical cancer cells^34^ and using pazopanib and paclitaxel synergy in anaplastic thyroid cancer^35^. In addition, the natural compound moscatilin, a bibenzyl derivative from the orchid *Dendrobium loddigesii*, has been demonstrated to induce apoptosis and mitotic catastrophe in human esophageal cancer cell lines^36^. The synergistic cytotoxicity of paclitaxel and MWE was achieved through the activation of PTEN, which converts PI(3,4,5)P3 to PI(4,5)P2 and, therefore, inhibits the formation of early endosomes, as revealed by EEA1 immunostaining (Fig. 3), under the influence of stabilization of the dynamic mitotic spindle by paclitaxel (Schematic summary in Fig. 6). The shortage of recycling endosomes led to the blockage of cytokinesis and induced mitotic catastrophe. To the best of our knowledge, this is the first report describing the contribution of mitotic catastrophe to apoptotic cell death by stimulating the activity of PTEN. A well-known tumor suppressor, inhibition of PTEN activity leads to the accumulation of PI(3,4,5)P3, abnormal activation of PI3K/Akt, unregulated cell growth, suppression of apoptosis, and increased tumorigenesis^37^. Our data present a novel and effective therapeutic option for bladder cancer treatment strategies via PTEN activation and inhibition of mitotic spindle disassembly to induce severe mitotic catastrophe and subsequent apoptotic cell death.

Analysis of the components of MWE by LC-ESI-MS-MS showed that the extract is largely composed of phenolic compounds and flavonoids. Among them, cyanidine 3-glucoside, chlorogenic acid as a component of the extract of *Zanthoxylum ailanthoides* Sieb and Zucc, and rutin have been demonstrated to induce apoptosis via G2/M arrest in breast cancer cells^38^ and colon adenocarcinoma cells^39^, respectively. A recent investigation of aqueous and organic extracts of mulberry leaves has demonstrated anti-proliferative activity through G2/M arrest, induction of apoptosis, and inhibition of topoisomerase IIα activity in the hepatocellular carcinoma HepG2 cell line^40^. Our results affirm the G2/M-arresting activity of mulberry extract and reveal that the underlying mechanism occurs by blocking cytokinesis via PTEN activation.

We combined MWE with other non-cytoskeletal chemotherapy drugs (paraplatin and 5-Fluorouracil) and found that MWE could not promote the killing of TSGH 8301 bladder cancer cells by these drugs (data not shown), suggesting that the cytokinesis-blocking effect of MWE functions in concert with the mitotic spindle-stabilizing activity of paclitaxel to strengthen the toxic action of this anticancer agent. The synergistic cytotoxic effect of paclitaxel and MWE has tumor specificity because another three cancer cell lines (including colon, liver and breast cancer cells) did not respond to the combined treatment to the same extent as the bladder cancer cells (data not shown). Although the molecular basis of such tissue specificity requires further investigation, the evidence presented in this study reveals a combination regimen using MWE to reinforce paclitaxel-induced mitotic failure in the treatment of bladder cancer.

## Materials and Methods

### Antibodies and reagents

The primary antibodies used in Western blotting and immunocytochemical analysis were as follows: antibodies against Cdc2 (#9112), phospho-Cdc2 (Tyr15) (#9111), Cyclin B1 (#4138), phospho- Cyclin B1 (Ser133) (#4113), Aurora A (#3092), phospho-Aurora A (Thr288) (#3079), phospho-PTEN (Ser380/Thr382/Thr383) (#9554) and Lamin A/C (#2032) were purchased from Cell Signaling (Danvers, MA); antibodies against Cep55, polo-like kinase 1(Plk1) (sc-17783) and phospho-Plk1 (Thr210) (sc-135706) were purchased from Santa Cruz (Dallas, TX); antibodies against Early endosome antigen 1 (EEA1) (GTX109638), phosphatase and tensin homolog (PTEN) (GTX101025), α-tubulin (GTX112141) and β-actin (GTX109639) were purchased from Gene Tex Inc (San Antonio, TX); antibodies against Caspase-3 (Pro and Active) (NB100-56708) was purchased from Novus Biologicals (San Diego, CA). Secondary antibodies Alexa Fluor 488 (557676) and FITC (562028) were obtained from BD Biosciences (Franklin Lakes, NJ). RPMI1640 medium, phosphate-buffered saline (PBS), trypsin-EDTA, penicillin-streptomycin mixed antibiotic, L-glutamine solution, HEPES and sodium pyruvate solution were purchased from Gibco/BRL (Gaithersburg, MD). Trypan blue solution was obtained from Thermo Fisher Scientific (Waltham, MA), and methylthiazolyldiphenyl-tetrazolium bromide (MTT) and 4′,6-diamidino-2-phenylindole dihydrochloride (DAPI) were purchased from Sigma-Aldrich (St. Louis, Mo). N,N,N’,N’-Tetramethylethylenediamine (TEMED), glycine, 1.5 M Tris (PH 6.8) and 0.5 M Tris (PH 8.8) were purchased from AMRESCO (Solon, OH), 10% SDS and 40% acrylamide from SERVA (Heidelberg, DE), ammonium persulfate (APS) from Pharmacia biotech (Piscataway, NJ), and NE-PER Nuclear and Cytoplasmic Extraction Reagent from Thermo Fisher Scientific (Waltham, MA).

### Extraction of aqueous fractions from mulberry fruit (MWE)

Mulberry (fruit of Morus alba L.) was obtained from the Miaoli District Agricultural Research and Extension Station in Gong-guan Township, Miaoli, Taiwan. The voucher specimen (76C001) is documented in the Council of Agriculture, Executive Yuan, Taiwan. Fresh mulberry fruit (54 kg) was frozen-dried and ground to obtain 5.4 kg of powder. The powder (100 g) was dissolved in 1000 ml of deionized water with stirring for 2 hr, and the extract was centrifuged at 3500 rpm for 20 min at 4 °C. After centrifugation, the suspension was frozen-dried. A yield of approximately 50% of the original dried powder weight was achieved. The lyophilized powder (MWE) was resuspended in distilled water to obtain the working concentrations and filtered (0.45 μm pore size) for subsequent use in cell cultures and in the animal model.

### High-performance liquid chromatography diode array detection and electrospray ionization mass spectrometry (HPLC-DAD-ESI-MS) analysis

Analysis of MWE components using HPLC-DAD-ESI-MS was carried out according to previous reports^41^,^42^, with slight modification. Samples were analyzed on a Surveyor HPLC system equipped with a diode array absorbance detector (DAD), scanning from 210 to 600 nm (Thermo Fisher Scientific Waltham, MA), and the chromatograms obtained were visually inspected at 278, 325 and 518 nm, respectively. LC analyses were carried out on a Symmetry C-18 column (Waters, Milford, MA). The mobile phase consisted of H_2_O (0.1% formic acid; solvent A) and acetonitrile (ACN, 0.1% formic acid; solvent B), applying the following gradient at a flow rate of 0.2 ml/min over 80 min at 25 °C: 0–3 min, 5% B isocratic; 3–40 min, 5–40% B; 40–60 min, 50–95% B; 60–75 min, 95% B isocratic; 75–80 min, 95-5% B. After passing through the flow cell of the DAD, the column eluate was directed to an LCQ Advantage Max ion trap mass spectrometer fitted with an electrospray (ESI) interface. Analyses utilized the positive ion mode (m/z M + H^+^) for the detection of anthocyanins and negative ion mode (m/z M − H^+^) for all other compounds. The mass optimization for the ion optics of the ion trap mass spectrometer was performed for chlorogenic acid m/z 353. MS/MS fragmentation was carried out with 35% energy. The identities of the compounds were obtained by matching their molecular ions (m/z) obtained by LC-ESI-MS and LC-ESI-MS-MS with literature data.

### Cell culture

TSGH 8301 cells line were obtained from Bioresourse Collection and Research Center (Taipei, Taiwan) and grown in RPMI 1640 medium supplemented with 10% (w/v) fetal bovine serum. Cells were maintained at 37 °C in the humidified atmosphere of a 5% CO_2_/95% air incubator.

### Cytotoxicity assay

Cytotoxicity was determined by the MTT reduction assay. Cells seeded in 24-well plates at a density of 3 × 10^4^ per well were exposed to paclitaxel and MWE for 24, 48 and 72 hr. After exposure, the cells were washed with phosphate-buffered saline (PBS) and incubated with 1 ml medium containing 100 μl MTT (5 mg/ml) for 4 hr. The viable cell number per dish was quantified by the ability of the living cells to reduce the yellow dye to a purple formazan product, which was solubilized in isopropanol and measured spectrophotometrically at 563 nm using an ELISA microplate reader (Bio-Rad Model 450).

### Cell cycle distribution analysis

TSGH 8301 cells were treated with MWE and paclitaxel at the indicated doses and for the indicated time periods. After treatment, the cells were fixed in 70% ice-cold ethanol-PBS at −20 °C after trypsin-mediated detachment from the culture plate. Thereafter, the DNA content was determined by staining with a solution containing 0.1% Triton-X 100, 20 μg/ml RNase, and 20 μg/ml propidium iodide (PI) at room temperature in the dark for 30 min. The cell cycle distribution was measured using CellQuest software with a BD FACS Calibur machine (Mountain View, CA).

### Western blot analysis

TSGH 8301 cells were seeded in a 10 cm^2^ dish at 1 × 10^6^ cells and cultured for 24 hr. The cells were treated with 0–1500 μg/ml of MWE and 3 nM paclitaxel for 24, 48 and 72 hr. The cytoplasmic and nuclear proteins of the cells were extracted by NE-PER Nuclear and Cytoplasmic Extraction Reagent (Thermo Fisher Scientific inc, Waltham, MA) and quantified using a Bio-Rad protein assay (Bio-Rad, Hercules, CA) with bovine serum albumin (BSA) as a standard. Each lysate (20 μg of protein) was resolved on denaturing polyacrylamide gels and transferred electrophoretically to NC membranes. After blocking with 5% nonfat dried milk in TBS-Tween 20, the membranes were incubated with primary antibodies at 4 °C overnight. The membranes were incubated with a horseradish peroxidase (HRP)-conjugated secondary antibody for 1 hr at room temperature. After being washed with TBST, the immunoreactive proteins were developed using an enhanced chemiluminescence kit (GE Healthcare Biosciences) and identified using the ImageQuant LAS 4000 mini.

### Hematoxylin and eosin staining

TSGH 8301 cells were seeded at 3 × 10^5^ and incubated on coverslips in 6-well plates for 24 hr and then exposed to paclitaxel and MWE for 24 and 48 hr. After treatment, the cells were fixed with 95% (v/v) alcohol for 1 min at room temperature followed by washing three times with purified water. The cells on the coverslips were stained with hematoxylin for 3–5 min to detect nuclei and then washed with ammonia solution for 30 s and 3 times with purified water. The cells were dehydrated by incubation for 30 s each in 70% (v/v) and 95% (v/v) ethanol and then stained with eosin for 1 min followed by immersion in 95% (v/v) and 100% (v/v) ethanol for 30 s each. The coverslips were immersed in xylene for 1 min and then mounted onto slides.

### Immunofluorescence and confocal microscopy

Cells seeded overnight on coverslips at 3 × 10^4^ cells were fixed in 4% paraformaldehyde on ice for 15 min, washed three times with PBS, and then permeabilized with 0.1% Triton X-100 in PBS for 15 min at room temperature. The cells were further blocked in 10% goat serum for 1 hour before the primary antibody was applied. After washing with PBS, the cells were incubated with an Alexa 488- or FITC-conjugated secondary antibody (Becton, Dickinson and Company, Franklin Lakes, NJ). Cell nuclei were counterstained with DAPI 32670 (Sigma Co., Steinheim, Germany). After extensive washing, the coverslips were mounted onto glass microscope slides, and the cells were viewed using a fluorescence confocal microscope (Zeiss, Jena, Germany).

### Immunohistochemistry of p-PTEN and fluorescent immunohistochemistry of Ki67

Expression of p-PTEN and Ki67 in paraffin wax-embedded tumor sections were detected by immunohistochemistry and fluorescent immunohistochemistry using the UltraVision Quanto Detection System HRP DAB (Thermo Fisher Scientific inc, Waltham, MA). Briefly, tumor tissue sections were adhered to slides and allowed to dry at 60 °C oven overnight. Slides were dewaxed in xylene, rehydrated in grading alcohol solutions and placed in PBS. Antigen retrieval was done by immersing sections in 0.01 M citrate buffer, pH 6.0, in a microwave 750W for 1 min, followed by rapid cooling (30 min). Slides were washed three times with PBS. Slides were incubated in 3% hydrogen peroxide to block nonspecific background staining due to endogenous peroxidase for 10 min. Then, nonspecific background was blocked by Ultra V Block and incubated for 5 min. After incubation (4°C, overnight) with the primary antibody phospho-PTEN (Ser380/Thr382/Thr383) (#9554) (Cell Signaling, Danvers, MA), Primary Antibody Amplifier Quanto was applied and incubated for 10 min. Slides were washed three times by PBS, incubated with HRP Polymer Quanto and kept in dark for 10 min. Finally, slides were incubated in 0.05% DAB for 1 min, rinsed in dH_2_O, counterstained with haematoxylin, dehydrated, and mounted.

After incubation (4 °C, overnight) with the primary antibody Ki67 (NB110-89717) (Novus Biologicals, San Diego, CA) with 2% BSA (Sigma Co., Steinheim, Germany), slides were washed and incubated with an Alexa 488 (Becton, Dickinson and Company, Franklin Lakes, NJ). Tumor cell nuclei were counterstained with DAPI 32670 (Sigma Co., Steinheim, Germany). After extensive washing, slides were mounted onto glass coverslips, and observed under a fluorescence confocal microscope (Zeiss, Jena, Germany).

### TUNEL method

For *in situ* detection of DNA fragmentation in paraffin-embedded tissue sections, the TUNEL method was performed using the *In Situ* Cell Death Detection Kit, POD (Roche, Basel, Switzerland). Briefly, tumor sections were adhered to silane-coated slides and allowed to dry at 60 °C oven overnight. Subsequently, slides were deparaffinized and rehydrated. Protein digestion was done by incubating tissue in 20 μg/ml proteinase K (Worthington biochemical Co., Lakewood, NJ) for 15 min at room temperature. Antigen retrieval was done by immersing slides in 0.01 M citrate buffer, pH 6.0, in a microwave 750W for 1 min, followed by rapid cooling (30 min). Subsequently, the slides were immersed in Tris-HCl, 0.1 M pH 7.5, containing 3% BSA and 20% normal bovine serum at room temperature for 30 min. TUNEL reaction mixture was added to slides and incubated at 37 °C in a humidified chamber for 1 hr. Tumor cell nuclei were stained with DAPI 32670 (Sigma Co., Steinheim, Germany). After extensive washing, the slides were mounted onto glass coverslips, and analyzed using an excitation wavelength in the range of 450–500 nm and detection in the range of 515–565 nm (green) by fluorescence confocal microscope (Zeiss, Jena, Germany).

### Xenograft model and treatment

Four-week-old BALB/c male nude mice were purchased from National Laboratory Animal Center (Taipei, Taiwan) and maintained for one week in a specific pathogen-free room with an irradiated 5058-PicoLab Mouse Diet (LabDiet, Inc., MO, USA) at 22 °C and 55% humidity with a 12 hr diurnal system. TSGH 8301 (1 × 10^7^ cells/mouse) cells were mixed with an equal volume of BD Matrigel (Becton, Dickinson and Company, Franklin Lakes, NJ) and injected into the right inguinal region of each nude mouse. When the tumor size reached approximately 450–500 mm^3^, the mice were randomly divided into 4 groups and received the following treatments: paclitaxel combined MWE with (12 mice), MWE alone (12 mice), paclitaxel alone (12 mice) and sterile deionized water (the control group, 11 mice). MWE (4 mg/kg) in sterile deionized water was given daily with a stomach sonde needle, and 147 nM/mouse of paclitaxel in sterile deionized water was injected into the peritoneum one time at the beginning of the treatment period. Tumor volumes were monitored using a caliper every week during the entire experiment. Tumor volumes were calculated from the following formula: tumor volume = major axis × (minor axis)^2^ × 0.52. All animal care and experimental procedures were carried out in strict accordance with the guidelines for the care and use of laboratory animals of Chung Shan Medical University, and approved by the Institutional Animal Care and Use Committee. This article does not contain clinical studies or patient data.

### Statistical analysis

Results are expressed as the mean ± SD, and data were analyzed by Student’s t-test or one-way ANOVA with post-hoc Dunnett’s test for significant difference with Sigmastat software (Jandel Scientific, San Rafael, CA). *p* < 0.05 was considered statistically significant.

## Additional Information

**How to cite this article**: Chen, N.-C. *et al*. Promotion of mitotic catastrophe via activation of PTEN by paclitaxel with supplement of mulberry water extract in bladder cancer cells. *Sci. Rep.* **6**, 20417; doi: 10.1038/srep20417 (2016).

## Supplementary Material

Supplementary Information

## Figures and Tables

**Table 1:** Identification of phenolic compounds in mulberry fruits by HPLC-DAD, LC-MS and LC-MS-MS.

Peak No.	*t*_R_	Compound	λ_max_ (nm)	[M + H]^+^/[M − H]^−^	MS-MS	Ref.
1	5.56	Quinic acid derivative^c^	233, 290	615/613		
2	38.02	Neochlorogenic acid^b^	327, 244	/353	191(100), 179(86), 135(8)	Vallverdú – Queralt *et al*., 2012
3	38.88	Cyanidine 3-glucoside^a^	236, 282, 515	449	287(100), 426(16)	
4	39.49	Cyanidine 3-rutinoside^a^	236, 282, 517	595	287(100), 449(52), 433(3)	
5	40.92	Chlorogenic acid^a^	328, 245	/353	191(100), 179(14), 173(1)	
6	42.09	Cryptochlorogenic acid^b^	337, 245	/353	179(100), 173(95), 135(23)	Vallverdú – Queralt *et al*., 2012
7	48.53	Rutin^a^	257, 233(sh), 357	/609	301(100), 300(67), 343(12)	
8	49.24	Isoquercitrin^b^	257, 233(sh), 356	/463	301(100), 300(61), 343(3)	Yang *et al*., 2012
9	58.93	Phenolic derivative^c^	279, 234	/311		
10	66.69	Phenolic derivative^c^	279, 236, 254	/279		

^a^The identification was further confirmed by an authentic compound.

^b^Compounds were tentatively identified according to mass spectra and matched data from the literature.

^c^Compounds were limitedly identified from mass spectra and UV-visible absorbance spectra.
