# Supplementary material for: Promotion of mitotic catastrophe via activation of PTEN by paclitaxel with supplement of mulberry water extract in bladder cancer cells
Source: Sci Rep. 2016 Feb 3;6:20417. doi: 10.1038/srep20417 (PMC4738303; doi:10.1038/srep20417)

Promotion of mitotic catastrophe via activation of PTEN by paclitaxel with supplement of mulberry water extract in bladder cancer cells

Nien-Cheng, Chen, Charng-Cherng Chyau, Yi-Ju Lee, Hsien-Chun Tseng, Fen-Pi Chou.

Supplementary Figure 1 Paclitaxel combined with MWE induced HT1367 and HT1197 bladder carcinoma cell death by arresting the cell cycle at the mitotic phase. (A) HT1367 and HT1197 cells were treated with paclitaxel (3 nM) alone or combined with the indicated concentrations of MWE for 24, 48 and 72 hr before being subjected to the MTT assay for cell viability. The data are expressed as a percentage of control (not treated) and presented as the means ± SD. One-way ANOVA with post-hoc Dunnett's test was used to calculate the *p* value for each dose treatment compared to paclitaxel alone ($^{+}$, p<0.05; $^{++}$ p<0.01) and between time points (*, p<0.05; **, p<0.01).

(B) HT1367and HT1197 cells were treated with paclitaxel (3 nM) and MWE (0-1500 μg/ml) and then subjected to cell cycle distribution analysis by flow cytometry at 48 hr.

A

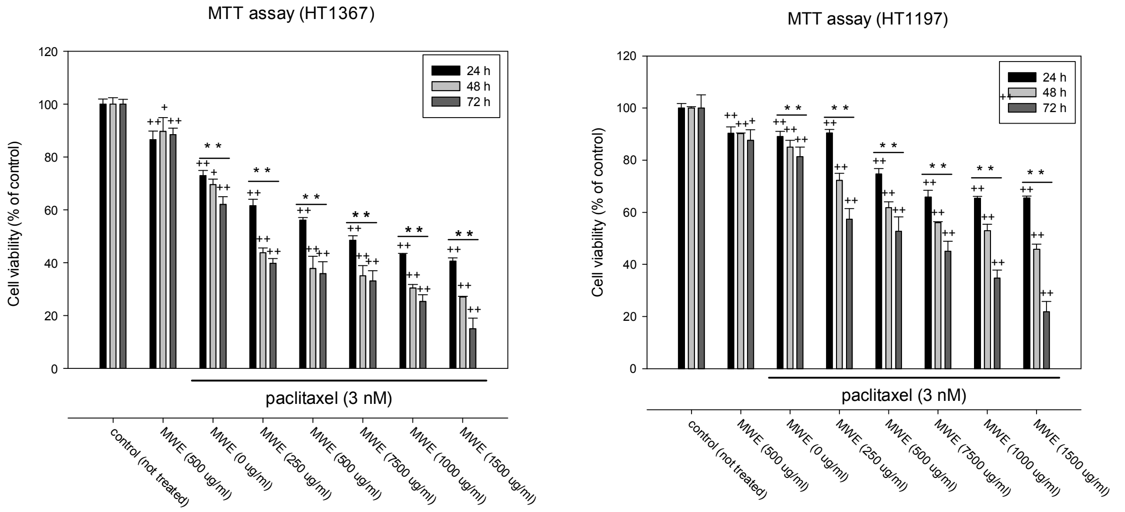

B

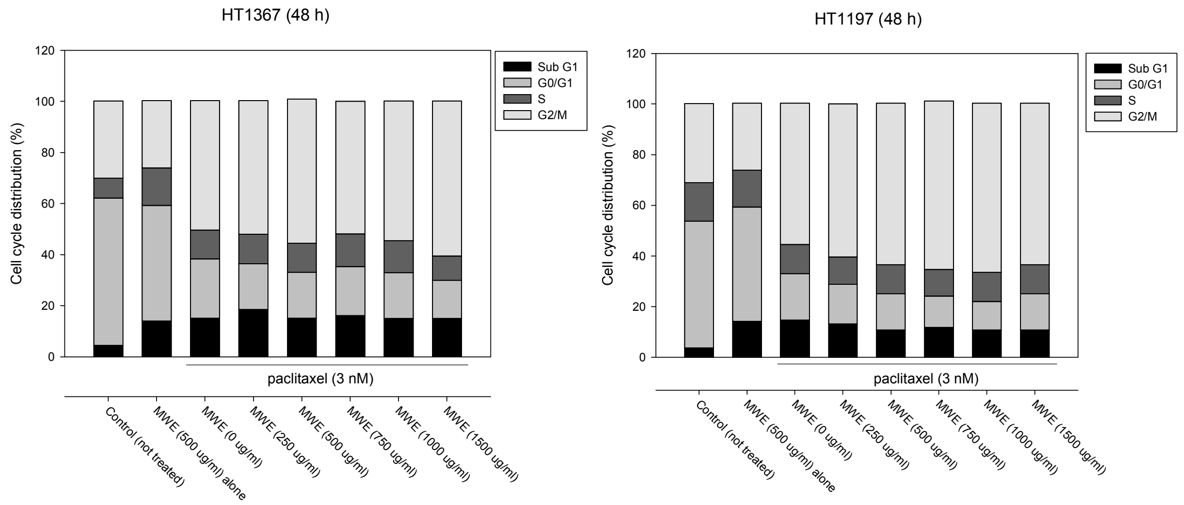

Supplementary Figure 2 MWE could not influence TSGH 8301 bladder carcinoma cell viability. TSGH 8301 cells were treated with MWE with the indicated concentrations of MWE for 24 hr before being subjected to the MTT assay for cell viability.

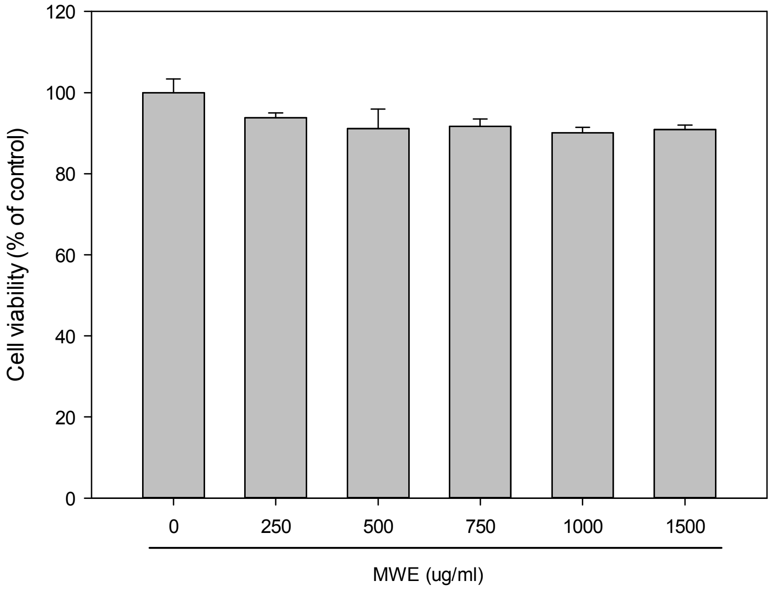

Supplement: Supplementary Information [file srep20417-s1.pdf]
